# Rapid Scale-Up of Screening for Early Detection of Sudan Virus Disease (SVD) in Healthcare Facilities (HCFs) during the 2022 Outbreak in Uganda

**DOI:** 10.1017/ash.2024.113

**Published:** 2024-09-16

**Authors:** Shillah Nakato, Judith Nanyondo, Martin Esagala, Maureen Kesande, Andrew Kwiringira, Ahumuza Noelyn Komugisha, Morris Aheebwa, Abdullah Wailagala, Elizabeth Katwesigye, Juliet Kasule, Isabella Fabens, Janelle Kibler, Elizabeth Bancroft, Doreen Nabawanuka, Paul Katongole, Mohammed Lamorde

**Affiliations:** Infectious Diseases Institute, Makerere University, Uganda; Ministry of Health, Uganda; Centers for Disease Control and Prevention; National Center for Emerging and Zoonotic Infectious Diseases, US Centers for Disease Control and Prevention, Atlanta, USA; Makerere University School of Public Health

## Abstract

**Background:** The Uganda Ministry of Health (MoH) and implementing partners instituted an infection prevention and control (IPC) response strategy during the Uganda SVD outbreak in 2022 that involved rapid enhancement of screening capacity at HCFs. Rapid scale-up of screening for infectious diseases, such as SVD, is critical for early identification and triage of suspected or confirmed cases in HCFs. We describe the rapid deployment of a multimodal IPC strategy implemented in response to the SVD outbreak and the resulting impact on screening measures at HCFs. **Methods:** We implemented a multimodal IPC strategy in HCFs from five high risk districts to improve screening practices from November 2022–January 2023. The strategy included training health workers (HCWs) identified as IPC mentors; establishing screening areas; and providing screening supplies and communication materials. The three-day training utilized an MoH standardized training package with didactic and practice sessions. The mentors then cascaded screening information and skills to other HCWs through onsite trainings and mentorships and established screening areas. Baseline and endline (3 months after baseline) cross-sectional assessments were conducted using the MoH IPC Assessment Tool adapted from the WHO Ebola IPC Scorecard. The five main screening parameters assessed included presence of ≥ 1 meter distance between screener and the person screened, availability of a functional handwashing facility and infrared thermometer, correct record of each person’s temperature, and appropriate referral process for those suspected of having SVD to holding areas. IPC capacity was measured through the summation of each of these parameter results and calculated as an overall percentage. IBM SPSS Statistics 20 software was used for data analysis and a paired t-test done to determine any significant findings between mean scores (percentage) at baseline and endpoint. **Results:** A total of 296 IPC mentors were trained, screening information was cascaded to 3,899 HCWs, and screening areas were established in 1,135 HCFs. Based on the screening results from the MoH IPC assessment tool, capacity improved from 44% (SD=37) at baseline to 67% (SD=34) at endpoint. Screening capacity improved from baseline to endpoint among level II and public HCFs from 33% (SD=35) to 60% (SD=35) (p < 0 .05) and from 54% (SD=38) to 76% (SD=31) (p < 0 .05), respectively. **Conclusion:** Rapid implementation of a multimodal IPC strategy was successful in enhancing screening capacity across Uganda’s HCFs during a SVD response, which is critical for early identification of infected patients to interrupt transmission. This multimodal approach should be recommended for future response actions.

